# Teachers’ contributions to the school climate and using empathy at work: implications from qualitative research in two European countries

**DOI:** 10.3389/fpsyg.2023.1160546

**Published:** 2023-04-20

**Authors:** Baiba Martinsone, Vilma Žydžiūnaite

**Affiliations:** ^1^Department of Psychology, University of Latvia, Riga, Latvia; ^2^Educational Research Institute, Vytautas Magnus University, Kaunas, Lithuania

**Keywords:** content analysis, educational psychology, empathy, focus group, professional well-being, school climate, student, teacher

## Abstract

This research aimed to reveal the factors that, based on their experiences, teachers consider important in their contribution to a positive school climate and in their relationship with students when it comes to using empathy. Teachers from two European countries—Latvia (*n* = 83) and Lithuania (*n* = 69)—participated in the research. Data were collected through focus group-based interviews and analyzed using latent qualitative content analysis. The results revealed the teachers’ shared understanding of the importance of communication and professional development. However, only Lithuanian teachers emphasized strengthening institutional values and other more collectivistic aspects; the answers of Latvian teachers were specific and suggested more of an individualistic attitude, like allocating personal responsibility to use appropriate instructional strategies. This study draws attention to contextual and cultural factors of teachers’ work and actualizes their educational needs.

## 1. Introduction

There is an increasing awareness among researchers, practitioners, and policy-makers that schools play an important role in promoting the mental health and well-being of both students and teachers. Social–emotional learning and academic learning have been recognized as equally important and related to the overall climate of an educational institution. The consistent development of students’ social–emotional skills and sustaining a positive school climate are expected to bring improvements at both the individual and the system levels. On the individual level, previous research provides evidence that students’ social–emotional competence is associated with academic achievement (e.g., Durlak et al., 2011; Corcoran et al., 2018), while higher social–emotional competence is positively related to the learning motivation and behavioral adjustment of children of both genders independently of their socioeconomic status, even at preschool age (Martinsone et al., 2021). Regarding the system level, the WHO’s Global School Health Initiative (WHO, 2020) addresses the importance of a positive psychosocial environment in fostering the well-being of all members of a school community. Research shows that positive social–emotional and behavioral outcomes are related to a positive school climate (e.g., Hough et al., 2017).

However, in the building of a positive school climate (including creating relationship-oriented and supportive learning environments, sustaining a student-centered pedagogy, inclusion, non-discrimination, and implementing social–emotional learning), the whole-school approach (as opposed to the separate activities of individual teachers) is of particular importance (e.g., Thapa et al., 2013; CASEL, 2015; Dusenbury et al., 2015). Research asserts the importance of sustaining whole-school efforts to improve the school climate and respect culture-specific norms, beliefs, and values (La Salle et al., 2021). The school climate as a multidimensional construct includes such aspects as norms and values, teaching practices, and interpersonal relationships (Cohen et al., 2009). Thus, educators are expected to be role models, implementing and sustaining productive communication at all levels (with students, parents, colleagues, and the administration), embodying values in their attitude and behavior, and even caring for the school’s physical environment.

For them to consciously contribute to a whole-school creation of a positive school climate, the work of researchers and practitioners should encompass a teacher’s personal characteristics, knowledge, and attitude, as well as their mental health and professional well-being. Research shows that teachers’ social–emotional competence, self-reflection capacity, and perceived well-being help to build more positive relationships with students, discipline them more effectively, and foster students’ growth (Hamre and Pianta, 2001; Jennings and Greenberg, 2009). Positive associations between the teacher’s well-being and students’ academic achievement (Herman et al., 2018) could be explained by the teacher’s capacity to adopt more supportive behavior and be successful in their role when they can manage their stress and sustain their personal well-being. Empirically examined positive relations have also been found between teachers’ and their students’ well-being in such domains as teachers’ psychological well-being and students’ satisfaction with the school, as well as teachers’ emotional exhaustion and students’ subjective health complaints (Bilz et al., 2022). Bilz et al. conclude that perceived teacher support has a mediating role in the relationship between teacher well-being and student well-being, proposing to proceed with research on what specific teacher behavior supports their students’ satisfaction with their school and, ultimately, students’ health. Consequently, consciously investing in a positive school climate and teachers’ and students’ well-being requires teachers’ knowledge, commitment, and skills to implement this knowledge in practice.

Teachers’ empathy enhances student learning. It is the degree to which the teacher works to deeply understand students’ personal and social situations, to feel care and concern in response to their emotions, and to respond compassionately with a focus on student learning. Teacher empathy is communicated to students through the teacher-student relationship within the teaching/learning of the specific subject (Meyers et al., 2019). Empathy plays a key role in social interactions and relationships, which are prerequisites for teaching and learning. Individuals can respond adaptively by perceiving others’ emotional states (emotional contagion), not becoming overwhelmed (emotional disconnection), and engaging with cognitive aspects of empathy (e.g., Grühn et al., 2008; Carré et al., 2013). The situation-appropriate response involves such aspects as an ability to regulate one’s own emotions, inhibit automatic reactions, and adapt one’s response accordingly. Previous research has concluded that empathy helps to cultivate social interactions, perceive them as more meaningful, and, specifically, that empathetic individuals can better accept negativity (Grühn et al., 2008). Consequently, this could help individuals to be more effective in complex social environments like educational settings. Grühn et al. also claim that empathy is associated with positive well-being, regardless of age.

However, the recent Eurydice report (European Commission, 2021) states that almost a quarter of European teachers report a negative impact of stress on their physical and mental health. The most frequently mentioned causes of stress in teachers’ work are administrative duties and workload, as well as responsibility for students’ academic performance, classroom management, and collaboration with parents. In relation to teachers’ professional well-being, external conditions such as workload, salary, and physical working conditions are most often at the forefront, with less emphasis given to aspects where they can promote their own and their students’ well-being. In their theoretical review, Gray et al. (2017) emphasize that both a positive school climate and teachers’ professional well-being have to be supported to ensure that students’ developmental and learning needs will be met. Nevertheless, for teachers to take responsibility for their own and their students’ well-being, they need to know exactly what to do.

Many programs, handbooks, and recommendations have been developed for implementation in teachers’ professional work. The “Learning to Be” project’s toolkit (Agliati et al., 2020) is an example of materials developed to equip in-service teachers with practically applicable instruction and formative assessment strategies to develop their students’ social–emotional skills. This toolkit provides teachers with teaching strategies applicable to lessons in every subject to foster students’ social–emotional growth. Another program addressing students’ mental health and teachers’ own well-being is the Promoting Mental Health at Schools (PROMEHS) curriculum (Grazzani et al., 2020). However, these tools are typically introduced to in-service teachers as an opportunity for professional development. At the same time, the vast majority of pre-service teacher study programs at universities, besides learning theories, instructional process, and evaluation technology, accentuate classroom management and didactics, with less focus on developing an educator’s introspective abilities, social–emotional skills, and their competence to sustain their own mental health in today’s educational settings that require constant changes to one’s teaching approach (e.g., Hipkins et al., 2018). There is a necessity to educate teachers early on to be capable of adapting to the changing learning environments, addressing the learning expectations and requirements of the student generation, implementing competence development, and using diversity productively (e.g., Gilbert, 2013).

The majority of conclusions on teachers’ well-being are based on quantitative measures, assessing such variables as teachers’ self-efficacy, exhaustion, and depressive symptoms (e.g., Capone and Petrillo, 2020). However, some recent research (e.g., Beltman et al., 2022) has addressed the importance of collecting qualitative data to add in-depth understandings to survey-based conclusions and to hear teachers’ voices. Therefore, to provide qualitative and culturally-specific data and to develop pedagogical implications for further teacher education, the present study aims to answer the following research questions through a qualitative analysis of interviews with teachers:

Q1: How do teachers contribute to a positive school climate?
Q2: How do teachers use empathy in their work?

The aim of the study was to reveal the factors that, based on their experiences, teachers consider important in their contribution to a positive school climate and in their relationship with students in the classroom when it comes to using empathy. This could help to identify teachers’ educational needs in order to establish and sustain environments facilitating their students’ academic growth and personal well-being.

## 2. Research methodology

### 2.1. Sample

The research participants comprised 152 teachers from two European countries—Latvia and Lithuania. Purposeful sampling was implemented in both countries. In both countries, the teachers participating in the focus groups were from schools involved in the “Teaching to Be” Project (each focus group included teachers participating in the project).

Different authors suggest different sizes for focus groups: Johnson and Christensen (2004) recommend 6–12 people, Langford et al. (2002) and Morgan (1997) suggest 6–10 people, and Krueger (2000) and Kuzel (1992) note that 6–9 people in a focus group is sufficient. The optimal size group is 8–10 people, depending on the demographic and research topic. Group sizes can be as many as 12–15, but it requires a strong moderator to facilitate that many people (Sim and Waterfield, 2019). Focus groups may engage up to 20 participants (Masadeh, 2012). Based on the researchers’ opinions about the size of the focus groups and the research design, the researchers in both countries determined that one focus group should have no fewer than 12 and no more than 20 participants.

#### 2.1.1. Latvia

Five focus groups were represented by teachers from five different schools. Two schools were from the capital city (Riga), and three were from other parts of Latvia. All schools were municipality-founded general education secondary schools (grades 1–12). The sample consisted of 83 teachers divided into five focus groups based on their respective schools. During the research, three focus group interviews were organized in each school involving the same groups of respondents. The groups of participants were as follows: Group A—15 participants (14 women, one man) from a town close to Riga; Group B—15 participants (14 women, one man) from Riga; Group C—18 participants (17 women, one man) from a regional city; Group D—19 participants (17 women, two men) from Riga; and Group E—16 participants (all women) from a regional city. Breaking the participants down by geographic area, 34 teachers were from Riga, 15 were from a town close to the capital city, and 34 were from regional cities. In terms of gender, there were 78 women and five men. The mean age of all five focus groups was 41 years old, and their mean teaching experience at school was 23 years.

#### 2.1.2. Lithuania

Five schools—pro-gymnasiums and gymnasiums—were invited to form teacher focus groups. These schools were chosen from big cities, small cities, and rural areas in order to represent the five biggest regions of Lithuania and reveal the multifaceted problems. Representatives of the school administration, parents of students, and students themselves did not participate in the focus groups, so the teachers were free to express their opinions, describe their experiences, and feel emotionally unstressed. Five focus groups with teachers were involved in the research, and each group was represented by teachers from one school: Group A had 15 participants; Group B had 10 participants; Group C had 15 participants; Group D had 14 participants; and Group E had 15 participants. In total, 69 teachers participated in the research. Breaking the participants down by geographic area, Groups A and B (25 teachers in total) were from major cities, Group C (15 teachers) was from a small city, Group D (14 teachers) was from a small town, and Group E (15 teachers) was from a rural area. In terms of gender, there were 53 women and 16 men. The mean age of teachers in all five focus groups was 44 years old, and their mean teaching experience at school was 21 years.

In Lithuania, a complete teacher’s education includes a bachelor’s (at a college or university) and a university Master’s degree in education studies. In Latvia, the compulsory basic education for teachers is a bachelor’s degree in pedagogy.

### 2.2. Data collection

Data were collected from September 2021 to February 2022 in both countries through focus group-based semi-structured interviews. This method was applied in order to explore teachers’ experiences, beliefs, and attitudes by using group processes to stimulate responses and gain insights through participants exchanging views and questioning and challenging each other (Scheelbeek et al., 2020). Following the recommendations of Nyumba et al. (2018) on how focus groups should be conducted, in both countries, every focus group interview took not less than 2 h and no more than 3 h.

A semi-structured focus group interview as a data collection method relies on asking questions within a predetermined thematic framework. However, the questions are not set in order or phrasing (Sim and Waterfield, 2019). It was used as an exploratory tool in this study autonomously by a researcher-moderator in both countries.

The qualitative empirical results presented in this article are related to four interview questions from a common questionnaire consisting of 18 questions asked to each of the five focus groups in the “Teaching to Be” project. These questions for focus groups covered areas relating to teachers’ professional role at school, namely school contextual components, learning to learn competencies, and teachers’ personal and social competencies.

The semi-structured interview questions asked in focus groups in both countries were related to research questions raised in this study:

Q1: How do teachers contribute to a positive school climate?

Interview questions:

What are the visible, easily recognizable characteristics of your school climate? Please describe the one that you treat as a key characteristic? Why do you say it is the main one among others? Please, provide specific example(s).
How do teachers contribute to a positive school climate in your school? Share your experiences. What expectations do you have regarding a better climate in your school?
Q2: How do teachers use empathy in their work?

Interview questions:

Share your experiences of how you use empathy at work. Share your experiences of best and worst examples from your professional practice.
How does empathy help you manage your emotions, feelings, and behavior in difficult situations at school? Please share specific examples from your professional practices.

### 2.3. Data analysis

Qualitative content analysis is based on the attitude that text is a data source with valuable information about concrete research phenomena (Kondracki et al., 2002). After grouping the text into subcategories and relating these to categories, the similarities and differences between specific content can be identified (Graneheim et al., 2017; Lindgren et al., 2020). Latent qualitative content analysis means interpreting what is hidden within the text. The researcher then needs to discover the implicit meanings of research participants’ experiences that are communicated in their interviews and what is implied rather than stated literally (Krippendorff, 2012). This methodology requires the researchers to be intimately involved in the analytical process and to use concepts to interpret and perceive data (Erlingsson and Brysiewicz, 2017).

Four stages of data analysis were applied, and each stage was performed several times to ensure the data’s quality and trustworthiness (see, e.g., Kondracki et al., 2002; Morse and Richards, 2002; Graneheim and Lundman, 2004; Bengtsson, 2016; Erlingsson and Brysiewicz, 2017; Kleinheksel et al., 2020; Lindgren et al., 2020):

1. Decontextualization with open coding: the researchers familiarized themselves with the qualitative data and read through the transcribed texts to obtain a sense of the whole before breaking the text into smaller meaning units. Each identified meaning unit was labeled with an inductively created code, which facilitated the identification of concepts around which the data was assembled into blocks and patterns. The researchers used a coding list with explanations to reduce changes during analysis to ensure reliability.
2. Recontextualization: the researchers checked whether all aspects of the content were covered in the list of meaning units in relation to the research questions. Then, the original text was re-read with the final list of meaning units. Meaning units that were not related to the research questions were excluded from the list.
3. Categorization with condensation: before the researchers created subcategories and grouped them into categories, the extended meaning units were condensed. This means that the number of words was reduced, but the essence of the meaning unit’s content was not lost. After the categorization process, the created categories were internally homogeneous but externally heterogeneous as no data fell into two groups and did not fit into more than one group of subcategories. All categories were rooted in the data from which they emerged.
4. Compilation: analysis began once the categories were established. A latent qualitative content analysis invited the researchers to immerse themselves to some extent in the data and to identify implied meanings in texts. The researchers chose particular meaning units for each category to be presented as quotations from the interviews.

Subsequently, both authors reviewed and discussed their separately identified categories before they reached a consensus. The common categories for each question were identified, and differences in categories of teachers’ answers were found and discussed.

### 2.4. Research ethics

Ethical principles, validity, reliability, and trustworthiness regarding the study’s research process, research design, methodology, and tools were assessed, and ethical permission to conduct the survey in both countries was received from the Committee for Educational Research Ethics, Educational Research Institute, Vytautas Magnus University (February 17, 2022, Protocol No. 5) and from the Research Board of Vytautas Magnus University (March 1, 2022, Protocol No. 17).

## 3. Results

To answer the first question about how teachers contribute to a positive school climate, two similar categories from Latvian and Lithuanian teachers’ responses were developed, namely, "developing professional capability” and “encouraging the self” (see Table 1).

**Table 1:** Content of similar categories in Latvian (*n* = 83) and Lithuanian (*n* = 69) teachers’ answers to the question “How do you contribute to a positive school climate?”

Category	Subcategory	Quotes from interviews (Lithuanian interviewees, *Latvian interviewees*)
Developing professional capability	Teacher autonomy	My autonomy is related to creativity. If I can act freely and creatively at school, then I can contribute to a better school climate and culture. Autonomous decisions, the opportunity to talk openly, and knowing that you will not be punished spreads the teacher’s wings of creativity.
		*I wanted to organize the Christmas party and be Santa. The administration supported the plan and provided me with all the attributes.*
	Teacher decision-making	The decisions I make both individually and when working in groups or teams are an important part of my professional creative development. When I am heard, I am listened to and my ideas are accepted; if they are not, there is a rational explanation. It is that decision-making that reflects my creative potential.
		*We are quite free to collaborate with colleagues. We can decide to have an idea for the topic of the lesson, for example, to lead the lesson together or to implement one topic in several subjects. There, in particular, our freedom is not restricted.*
	Continuous learning	The continuous learning of a teacher is an inspiring example for students, their parents, and the entire school community. A teacher’s mission is to help students and the general public realize that learning never stops if you are open, flexible, and proactive. This always contributes to the development of creativity, and this allows you to work in various groups, teams, and projects, make various professional choices in life, and discover hobbies. I think that’s how I contribute to the idea that students need to love learning.
		*The school administration organizes supervisions.*
		*The school participates in all available projects in Latvia. It promotes professional growth, and each of us gives something to each other—experience, reflections.*
Encouraging the self	Adapting to innovations and changes	I think there is resistance only because there is a heavy workload and a lack of information—how it is useful for the teacher, the student, the school. Therefore, talking with teachers and making decisions about innovation together is essential. However, we cannot always manage the situation because a significant part of innovations comes from outside, from the ministry, from politics—then, whether you like it or not, you have to implement it. And flexibility is necessary both as a person and as an expert on the subject. And for that, you need to motivate yourself, enable yourself to constantly ‘be on the wave’ by learning, observing, adapting to the context, situations, circumstances, but not forgetting your professional role and mission.
		*The feeling that no one is really listening to us since the new education reform; there is a lot of uncertainty and a lack of information in the reform itself. In that uncertainty, the teacher is held hostage, and this leads to burnout.*
	Improving competencies	When I think about competencies, the first thing that matters to me is my educational subject—to follow it and to know all the innovations. This is related to didactics—to teach in such a way that students understand and learn, and their learning and achievements can be seen as the results of their own efforts. I dedicate time and effort to learning—qualification improvement courses, participation in seminars, conferences. I also spend time working with students, talking to them, trying to understand them. And this is also a kind of improvement of competencies. So alive, so realistic.
		*The school’s administration recommended that I also become the English teacher. I was supported during my studies and received study leave before graduating.*
	Leading in multiple contexts	I see the existence and need for my leadership in many different ways. It is difficult within my subject, teaching, student learning, interactions among fellow teachers, and decision-making from the classroom to the whole school level. But that teacher leadership must be allowed and visible, supported and positively received. It is important that the teacher is seen as learning, able, and capable. I assume leadership in various contexts—organizing excursions for students and fellow teachers, initiating events, talking with students’ parents, solving students’ educational and social problems. I must occupy multiple roles at once.
		*I try to get a student who is an authority in the class to be on my side. If successful, the student in the class also sets the tone and helps maintain order. Then it’s easier. I’m not alone then, we are a team.*

Developing professional capability includes the following three subcategories: (i) autonomy, which teachers relate to acting freely and creatively at school; (ii) decision-making, which participants connect to cooperation with colleagues while working in groups; and (iii) continuous learning, which teachers link to professional growth and the opportunity to engage in various activities with colleagues while being a role model for students and the school community.

*Encouraging the self* consists of three subcategories: (i) adapting to innovations and changes, which relates to teachers’ ability to adjust to rapid changes that cause them a sense of uncertainty, and since these changes will occur for a long time, flexibility in attitudes to professional activity is essential; (ii) improving competences, which reflects the variety of learning methods teachers use—studying in higher education institutions, learning in formal courses, and daily experiential learning while working with students in the classroom; and (iii) leading in multiple contexts, which means leadership in a variety of contexts and formal and informal activities.

The results show specific differences in experience-based perceptions among Latvian and Lithuanian teachers regarding their contribution to a positive school climate (see Table 2).

**Table 2:** Content of different categories in Latvian (*n* = 83) and Lithuanian (*n* = 69) teachers’ answers to the question “How do you contribute to a positive school climate?”

Category	Subcategory	Illustrative quotes from interviews
Latvian teachers		
Building personal connections	Personal interest	I am interested in how the students have spent their holidays.
		I really try to memorize my students’ names and use them both in class and when meeting students in the hallway or outside school. I see that some children are pleasantly surprised that I remember their names.
		I am really interested in what happens to students in their free time. I always ask how they are doing, and they are not afraid to ask what I do either. When I say that I like to run and ride a bike, they are amazed that we do the same things as they do.
	Informal activities	We organize common parties, events, and trips.
		We organized the “Night at school” event—students prepare dinner in the school cafeteria, go on a night tour of the school, sleep in class, etc. Such activities strengthen the relationship between teachers and students and also between students.
		A positive climate is also created by small groups, where people come into contact more often and create a microclimate. We talk about everyday things and feelings.
Problem-solving	Team-building through professional relationships	Working groups for teachers representing the same subject area.
		Collaboration between subject teachers—parallel class teachers work together to develop tests and uniform requirements and exchange experience and teaching materials.
	Access to administration	As you walk through the hallway, the doors of the administration staff are always open; you can enter and ask questions.
	Sustainability of the team	Twenty-five of us—teachers and administration staff—have graduated from this school.
	Reframing conflicts	When there is a conflict in my class, I always ask, “How can I help you?” instead of asking who is guilty. I have seen that my students now use this approach independently in their conflicts.
	Debriefing	I went to the vice-principal and told her how bad everything was. She let me complain, but she also helped me to calm down.
	Regular meetings	Every Tuesday, we have a small information meeting—it keeps you disciplined, you find out the news, colleagues remind you what has been forgotten and what needs to be done; it motivates you and makes you feel informed and inside the process.
Physical environment	Coziness	Our school is cozy. It is a cozy environment. I like the layout of the school, the warm colors in the classrooms, the newly renovated premises. We create our own environment; for example, we once even put curtains over the windows.
	Providing structure	We have pictograms to regulate the order in which people speak.
Lithuanian teachers		
Strengthening institutional values	Respect	Respect each other in the classroom and at school. Between the teacher and student, teacher and teacher, and teacher and school administration, in the whole school community. Through real actions. This must be reflected everywhere – in conversations, in correspondence (emails and comments), in our decisions. There is still a lot of work to be done to learn to hear each other, to listen, to cooperate, to criticize, to make decisions, to solve problems. We can maintain a positive atmosphere in the school only if we all focus on fostering mutual respect.
	Dignity	Dignity is often declared, but it is still difficult to find it in actions and cooperation. Although there are positive signs. Here, the teacher plays an important role through their example. Therefore, the responsibility is great.
	Humaneness	Humaneness is reflected in all the teacher’s actions—from assessments to conversations with students. This does not mean pandering or trying to please the student; it is closer to social sensitivity, being aware of the situation, the context, and the person.
	Sustainability	Sustainability is an important value of the school—to live in peace with nature, to contribute to a school’s ecology physically, morally, and socially. It is a complex learning process where a single teacher cannot do much. Therefore, strengthening the school administration and mobilizing the entire school community plays an essential role here.

Latvian teachers view personal connections, solving problems, and maintaining the school’s physical environment as important in maintaining a positive school climate. Teachers accentuated the importance of building positive relationships with students and fellow teachers while organizing informal activities and implementing team-building. They also emphasized the importance of accessible administration, conflict resolution, regular team meetings, the opportunity to debrief in a situation of increased stress, and attention to the school’s physical environment.

Lithuanian teachers talked about supporting institutional values by highlighting mutual respect, dignity, humaneness in teacher-student, teacher-teacher, and teacher-administration relations, and sustainability as the necessary characteristics of a positive school climate. They see meaning in strengthening the school community, practicing social sensitivity in relation to the situation, context, and person.

There were also similarities in what teachers from both countries said about using empathy at work. To answer the second question about how teachers use empathy in their professional work, four similar categories from teachers’ responses in the focus groups were developed.

*Teacher-student communication* includes the following four subcategories: (i) teachers’ attentiveness to students, which is based on regular observation and building awareness of students’ needs; (ii) talking sincerely with students; (iii) maintaining equivalence in mutual conversations; and (iv) maintaining respect and dignity in mutual conversations.

*Supporting students* consists of the following four subcategories: (i) helping students to solve learning difficulties; (ii) listening to and hearing students’ opinions’; (iii) teachers’ attentiveness to a student’s family context, which takes into account a wider ecosystem of the learning process; and (iv) providing targeted help through an individual approach to every student in need.

*Teacher-parent communication* comprises the following three subcategories: (i) involving parents in their child’s learning by establishing relationships in order to facilitate students’ learning motivation and achievements; (ii) providing targeted counseling to parents to support their child’s learning and well-being; and (iii) finding a solid basis for consensus with parents and preventing problems when a conflict could arise.

*Teacher-teacher communication* involves the following two subcategories: (i) mutual professional support, providing a secure base for teachers’ professional growth, and (ii) mutual professional respect and dignity as a fundamental aspect of a teacher’s work and a channel through which to use empathy (Table 3).

**Table 3:** Content of similar categories in Latvian (*n* = 83) and Lithuanian (*n* = 69) teachers’ answers to the question “How do you use empathy in your work?”

Category	Subcategory	Quotes from interviews (Lithuanian interviewees, *Latvian interviewees*)
Teacher-student communication	Teachers’ attentiveness to students	The teacher’s attentiveness at every step, in every context, in every situation. Do not be little, do not compare with others. This allows the teacher to maintain a good atmosphere in the classroom. By paying attention, the teacher shows empathy.
		*If I see that children are exhausted, I will try to find a more interesting and slightly easier task to do in my lesson.*
	Talking sincerely with students	Sincerity in the relationship with students is best reflected in conversations. And there are all kinds of these—both problematic and unproblematic, simply talking about an experience among themselves. Without sincerity, the relationship is cold, insensitive. Therefore, it is an important quality that does not need to be hidden and suppressed. It is not like or dislike. This is heartiness.
		*We come to work to see smiling faces and feel welcomed.*
	Maintaining equivalence in mutual conversations	Equality in conversations between teachers and students is essential. It is not the quality of frivolity. This is another characteristic of respect without crossing moral and ethical boundaries. It is an opportunity for both the teacher and the students to grow, develop, and show their best sides.
		*I speak about my emotions openly. I said that I was so overwhelmed that I had not had time to prepare for the lesson. The students were empathetic, and our relationship improved after this lesson.*
	Having respect and dignity in mutual conversations	Mutual respect and maintaining dignity in conversations between teachers and students is essential. We learn this every day. This is the path to intelligence. This is what I always strive for and want. The students feel it. And I feel their efforts. It is inseparable from ethics and morality and personal responsibility for one’s words and actions.
		*We respect the one who speaks.*
Supporting students	Helping students to solve learning difficulties	Support for students is considered very direct when targeted to solve learning problems. This happens in various ways—by recommending, talking, showing, presenting examples, discussing, giving reading suggestions.
		*I handed out white sheets of paper to the children, and every time they wanted to say something that was not relevant to the lesson, they wrote it on that sheet. It helped alleviate the students’ emotions that prevented me from leading the lesson.* *We have a rule that a test can be rearranged within two weeks, but if I see that the student is overloaded and does not have the opportunity to rearrange the test now, we can agree that they can come to it later.*
	Listening and hearing to students’ opinions	Listening to students’ opinions and attitudes means showing support for them. Not only respect. It means speaking and talking, expressing visions and sharing them with them, and learning to think critically and politely to talk about all kinds of topics. No matter whether they are hard or difficult.
		*I often ask students, “What do you think?” It’s easier to emphasize that I may not know something and I can ask for help.*
	Being attentive to a student’s family context	Students are related to their families and therefore to their family context. It is always individual, authentic, sensitive. Therefore, my attention to the student’s family context is an integral part of teaching and a component of my private ethical communication with them.
		*I’m too empathetic. I accidentally fell into the role of a student’s mother. I think about what I would do if I were this student’s mom.*
	Providing targeted help for students	Targeted support for students is important. Not for everyone, but for individuals. I understand this very well, and I always help in a targeted way.
		*I usually have a coloring book in my bag. There have been situations where a student in the class has been aggressive, dissatisfied. I suggest to this student that they paint a page of their choice in this coloring book. The student usually calms down fairly quickly and can continue working in the classroom.*
Teacher-parent communication	Involving parents in their child’s learning	It is not easy to consciously involve parents in their child’s learning. But it is necessary. Their involvement is directly related to the child’s learning motivation and achievements. Then the child does not feel alone and lost. A teacher is not enough. Therefore, we spend a lot of time with the children’s parents: we talk, argue, discuss, reach a consensus, and talk about their obligations to the child and their future.
		*I call parents not only when a student has done something wrong but also when they have behaved well. Usually, the parents are surprised, and the child really tries to behave well over the next few days.*
	Providing targeted counseling to parents	Targeted consultations for parents regarding the child’s learning, well-being, emotion management, and behavior are common topics. I myself learn a lot and share my experiences with them. The most important thing for me is the child. They should love learning and feel good so that learning becomes daily hard work but without coercion. Parents must be involved. Therefore, I provide targeted consultations and devote a lot of time to jobs. I think this is one of the prerequisites for my success in working with students.
		*After receiving a low grade on a test, the student cried. I said I’d call her mom and explain everything, that she had nothing to worry about.*
	Finding a solid basis for consensus with parents	Finding a solid basis for consensus-based decisions with someone’s parents is not easy, but in many cases, it is possible. I spend time and effort on this. But I do it with love and openness. Because I’m doing it for the sake of the child. And in those conversations with parents, we learn both sides—we listen, we hear, we are patient and open, non-judgmental, so we grow and learn from each other.
		*When there are conflicts, I try to keep the peace, do not raise my voice, do not look for the culprit, and continue the conversation calmly. Then the students or parents also remain calmer. I focus on the current situation, ask questions and try to gather as much information as possible.*
Teacher-teacher communication	Mutual professional support	Mutual professional support among teachers is one of the prerequisites for survival in the teaching profession. It is related to methodological solutions, solutions to problems in teaching and learning, creative initiatives, preparation for lessons and competitions, and countless other aspects. It requires teachers’ kindness, flexibility, and openness. It is part of the culture of communication formed by teachers and the school administration. You will feel safe as a professional if such a culture is supported in the school. I have been living in this culture for two decades. This is professional happiness.
		*A sense of security is provided by colleagues, and they also provide support.*
	Mutual professional respect and dignity	Mutual professional respect and dignity are inseparable from a teacher’s professional well-being in school. These include self-esteem, self-worth, mutual trust, openness, flexibility, friendliness, honesty, and selflessness. There is no place for egoism and egocentricity and narcissism. Ethics and morality, responsibility and duty are what is required.
		*I know that if I am sick, then a colleague will be obliged to do my work too. This is why I keep doing what I can for as long as I can, and I always bring some sweets when a colleague has helped me.*

Several country-specific differences emerged from the qualitative data regarding teachers’ use of empathy in their professional work.

Based on the Latvian teachers’ answers, two different categories were developed, namely, *Classroom instruction* and *Teacher’s personal responsibility* to create space for empathy. Latvian teachers use empathy through the content of the subject (e.g., history), classroom management strategies, and adapting their teaching methods and classroom management to their students’ learning needs. In terms of the teacher’s personal responsibility, Latvian teachers use empathy by creating a positive mood in the classroom and at school and changing expectations and perceptions regarding students’ learning process and needs.

Lithuanian teachers use empathy by supporting students and communicating with students’ parents and fellow teachers. They also mentioned giving timely feedback to students, the attentiveness of students’ parents in listening to their opinions and expectations regarding the child’s learning, and openness to fellow teachers in solving problems and making decisions as instances of using empathy in their professional work (Table 4).

**Table 4:** Content of different categories in Latvian (*n* = 83) and Lithuanian (*n* = 69) teachers’ answers to the question “How do you use empathy in your work?”

Category	Subcategory	Illustrative quotes from interviews
Latvian teachers		
Classroom instruction	Using subject content	There are so many events in 20th-century history that cannot be talked about without empathy, such as the Holocaust and World War II. When I have to talk about these historical events, I can relate these events to my family’s life story and to the experiences of the students’ families.
	Using classroom management strategies	I try to ask students how they feel. Then if some say that something is wrong, I ask what could help them. If, for example, a child says that silence in the classroom would help him, the whole class tries to respect it more. The classes I remember asking them how they feel usually go better because then I can remind them, “Hey, we agreed that there would be silence in the class.”
	Adapting teaching methods to students’ learning needs	I try to watch the kids for a full school day. The aim of the observation was to see how children behave from 1 h to another and how the child endures those seven hours. I noticed that it is difficult for students to learn if the same teaching methods are used in several lessons, such as using only workbooks. The child no longer follows the lesson because he is tired and uninterested, even though the content is different.
		I evaluate the emotional mood of the students at the very beginning of the lesson, how I can keep their attention on the lesson, and I try to adapt. I adjust to the overall emotional state of the class.
Teacher’s personal responsibility	Creating a positive climate	The teacher creates a positive mood; if they come to work positive and smiling, it simply sticks to others.
	Changing expectations	It was difficult for me to understand and accept that my child has difficulties with learning, especially Latvian. A child growing up in a Latvian family does not understand the grammar of the mother tongue! It was a shock, a blow, like a heart attack! And I am a Latvian language teacher. But now, thanks to my child, I understand why my students may not understand much better. I used to be very categorical—I could not understand how a child whose mother tongue is Latvian does not know grammar. I could understand why this was the case for children of other nationalities, but not Latvian. Now, I think differently. I am no longer so categorical. I do not assume that a child understands grammar.
	Changing perceptions	At the moment, when someone is very disturbed in class, I try to change my perception—I turn on the love button and give an inner signal to myself that this is a very nice boy. I look at him and give myself a signal that he is nice. I seem to deceive myself, then my emotions subside, and I can react more calmly.
Lithuanian teachers		
Supporting students	Providing timely responses to students’ queries	Timely feedback to students is important and necessary. As it is for all of us adults. Especially when it is related to learning, learning tasks, results, and problems. I understand this, and I try very hard to provide answers to students’ queries promptly.
Teacher-parent communication	Listening to parents’ opinions and expectations regarding their child’s education	Parents are used to demanding. Sometimes I listen to them, and it’s hard for me to understand what they want for the child. Therefore, I talk to parents a lot, I try to be their supervisor so that they reflect and consciously focus on their child’s learning needs and expectations. I do not think the parents’ expectations and wishes for the child’s education are higher than the child’s. And the child’s wishes and expectations must be nurtured and developed by teachers and parents together. The child must also participate in this. It is a complicated process. But very necessary.
Teacher-teacher communication	Being open while solving problems and making decisions	Teachers need mutual openness to make decisions transparently and clearly. Open conversation, openly expressed emotions, openly communicated feelings. When we learn to do this, we want to go to work, we work motivated in groups and teams. We can make difficult decisions and be calm about them because they are clear, ethical, and moral.

## 4. Discussion

### 4.1. How do teachers contribute to a positive school climate?

Both Latvian and Lithuanian teachers said that they contribute to a positive school climate through their capability of acting autonomously, making decisions individually and with fellow teachers, learning continuously, and being role models to students and the school community. These results show that the school climate is not just an individual experience. A positive school climate fosters teachers’ professional development and students’ learning, and vice versa—teachers’ professional development contributes to a positive school climate. The school’s norms, values, and expectations can support the school community in feeling socially, emotionally, and physically safe. Other authors have suggested that this could mean that teachers form and nurture an attitude that emphasizes the benefits of learning (Cohen et al., 2009).

This research aimed to reveal factors that teachers consider important in their contribution to a positive school climate and in their relationship with students when it comes to using empathy. Only Lithuanian teachers emphasized strengthening institutional values and other more collectivistic aspects. Conversely, the answers of Latvian teachers were specific and suggested more of an individualistic attitude. Taken together, the attitudes of Latvian and Lithuanian teachers form an optimal model that integrates individual and collective community aspects. However, they are both specific to the context of the school. A positive school climate is a combination of personal, communal or collective, and contextual factors that increase prosocial interpersonal relationships among students and decrease problem behaviors (Zych et al., 2019). Contextual factors influence teachers’ decision-making regarding teaching and learning since social interactions, culture, society, technology, and other contextual factors are learning assets. Students come to class with different learning standards and attitudes toward learning. This is related to biological factors, family contexts, cultural aspects, socioeconomic dynamics, and other assets affecting students’ learning. Thus, when teachers discuss their expectations with students regarding their learning and outcomes, they should take into account all these factors.

The pedagogical environment designed and facilitated by the teacher plays a role in students’ creative development, which builds the school’s positive climate (Anderson et al., 2021). However, despite speaking about the same aspects of building a positive school climate, only Lithuanian teachers talked about creativity; Latvian teachers did not highlight this aspect in their answers. It is possible to speculate that this is because there is a difference in teachers’ pre-service education. In Lithuania, a complete teacher’s education involves obtaining a Master’s degree, which includes developing an understanding of educational philosophy. In Latvia, the minimum pre-service education is a bachelor’s degree in pedagogy or any other specialty together with a brief 72-h course in pedagogy. Consequently, teachers’ education in Latvia has been more practically oriented until now, addressing classroom management, didactics, and assessment. Only during the last several years have such courses as social–emotional learning in schools been introduced to pre-service teacher education programs. Another explanation could be that in Latvia, creativity is attributed more to students’ performance than teachers’ professional work.

Encouraging the self is the other category that emerged from the answers of teachers from both Latvia and Lithuania. In building a positive school climate, teachers encourage the self by adapting to innovations and change and improving competencies and leadership in different contexts. These findings are consistent with the attitude of Kachnic and Berkowitz (2022), who note that teachers play a vital role in offering stability, a sense of belonging, and social relationships that can promote their learning to students. Teachers accomplish this by being flexible to innovations and change, open to other attitudes, critical of stereotypes, and self-critical, challenging their professional comfort. Teachers from both countries also emphasized the negative side of rapid changes in education that could diminish their capability to invest in building positive learning environments (i.e., by facing continuous changes, the teachers’ approach could become more about surviving than thriving). However, teachers’ professional and/or competence development is a crucial factor that contributes to a positive school climate. Teachers increase their mindset and their abilities to support and address the social and emotional needs of students and themselves through competence improvement (Durlak et al., 2011). A positive school climate and teacher leadership have both been shown to have beneficial effects on student achievement. Teacher leadership implies that teachers hold the key role in teaching and learning. In this way, teachers are given the professional power to be a part of creating a positive climate in schools. Thus, teachers’ self-encouragement through their leadership becomes an important component of a school climate (Gningue et al., 2022).

Several country-specific differences emerged when the teachers spoke about their contributions to a positive school climate. Latvian teachers accentuated building personal connections, solving problems appropriately, and maintaining their school’s physical environment. Teachers shared their experiences of building positive relationships through expressing personal interest in their students’ learning and achievements, echoing finding of Eccles (2008) that building strong personal relationships is an important part of creating a positive school climate. Latvian teachers also saw organizing informal activities to mobilize the school community and providing team-building activities with fellow teachers through professional relationships as part of creating a positive school climate. This is consistent with the conclusion of a recent systematic review that social relationships play a central role in predicting teachers’ well-being, which consequently influences their teaching quality (Hascher and Waber, 2021). These results also support the research of Greenway (2017), who shows that a well-maintained school environment improves students’ achievement on standardized tests and is linked to increased teacher commitment.

For Latvian teachers, the physical environment, accessibility of the school administration, professional teamwork, and resolving conflicts are inseparable parts of the school climate. These answers align with the conclusions of other researchers that the construct of a school climate is multidimensional (see, e.g., Thapa et al., 2013; La Salle et al., 2021) and involves such aspects as perceptions of peers’ and adults’ interpersonal relationships, learning environment (including teachers’ expectations and material resources), and perceptions of physical and emotional safety. Consequently, a supportive school climate can sustain teamwork and conflict resolution, thus supporting physical and emotional safety. This means that the school climate is not the final outcome but a continuous and dynamic process of improvement. Effective school climate improvement efforts involve students, parents, teachers, and the whole school community (Cohen et al., 2009).

Lithuanian teachers accentuate institutional values—respect, dignity, humaneness, and sustainability. For them, mutual relations, cooperation, and various activities are the responsibility of every member of the school community. They are convinced that the school community only supports the institutional culture and contributes to a positive school climate by implementing such values in practice, i.e., in their real-life relationships. Since the school climate refers to various values that are key to the development of a student’s personality (Gálvez-Nieto et al., 2022), Lithuanian teachers emphasize communality. Communality is related to more positive behavioral strategies and a teacher’s consistent enforcement of rules, which is associated with a more positive school climate (Mitchell and Bradshaw, 2013).

### 4.2. How do teachers use empathy in their work?

Both Latvian and Lithuanian teachers declared that they use empathy through communication with students, teachers, and parents. In teacher-student communication, empathy means attentiveness, kindness, respect, and equity. Teachers use empathy by supporting students regarding their learning and personal difficulties, respecting their family context, and taking into account their personal opinions. Teachers also provided examples of using empathy in communication with parents by involving them in their child’s learning, providing counseling, and striving for consensus. Teachers also use empathy while communicating with fellow teachers by providing mutual support on the basis of respect and dignity. These results match other research-based considerations (Gair, 2009; Gutsell and Inzlicht, 2010) that empathy builds a positive classroom culture, strengthens the community, develops soft teacher leadership, and prepares students to be leaders in their community through the use of teachers as role models. A teacher’s soft leadership is related to soft skills and is a process involving striving for teaching/learning goals; having a persuasive influence on students and fellow teachers and forming effective teams; negotiating with others with a win-win attitude and valuing others’ failures for learning from them; supporting students and fellow teachers and motivating them for learning, achievements and success; and recognizing others’ contributions in implementing teaching/learning goals (Rao, 2017).

Several country-specific differences were found regarding teachers’ use of empathy. Latvian teachers consider that they use empathy through a subject’s content by using positive classroom management strategies and adapting their instructions to the emotional mood of students in a classroom. Latvian teachers pointed out that it is their personal responsibility to create a positive mood and change dysfunctional beliefs and attitudes. The teacher’s capability to manage classroom instruction strategies for students’ growth has also been recognized in previous research (Ferreira et al., 2020). Considering the emotional state of students helps to identify, plan for, and implement preventative techniques to encourage positive student behavior, minimize disruptive behaviors in the classroom, and support their learning motivation (Banks, 2014). Nevertheless, the Latvian teachers spoke more about their personal responsibility regarding their use of empathy rather than waiting for collegial or parental support. This is the opposite of their answers about building a positive school climate, when they exclusively spoke about the importance of building relationships. Thus, one could speculate that empathy is perceived as a personal trait, naturally arising in teachers’ professional work, rather than as an ability that must be developed in teachers’ training.

Lithuanian teachers apply empathy by responding promptly to students’ queries and questions. These results support the attitude that learning is an emotional process (Banks, 2014). When students are excited at learning a new skill, they can experience negative emotions about mistakes and situations when they are misunderstood. Thus, operatively provided feedback from a teacher can foster positive emotions in the classroom and serve as a premise for motivation to learn (Greenway, 2017). Lithuanian teachers also emphasized the role of fundamental values (respect, dignity, and kindness) and the importance of collaboration with colleagues by relating it to using empathy at work. This means that empathy is a value desired, supported, and practiced by teachers in different aspects of their professional work. Therefore, Lithuanian teachers’ answers represent the attitude (Çakiroğlu et al., 2012; Gálvez-Nieto et al., 2022) that a school climate encompasses the experiences of school community members while teaching and learning, building mutual relationships, and capturing communal beliefs and attitudes. Implementing this whole-school consensus requires individual action, empowerment, awareness, and courage. Therefore, the individual contribution of the teacher to the development and improvement of a positive school climate, emphasized by Latvian teachers, remains relevant (Greenway, 2017).

In short, Lithuanian teachers associate empathy with their community, society, and institution, providing more general statements, while Latvian teachers realize empathy through personalized professional expertise, emphasizing their own individual responsibility. These differences in their answers can be attributed to collectivistic and individualistic cultural orientations. Research in individualism and collectivism is usually related to cultural orientations, defined by whether more emphasis is placed on the individual or group, itself usually attributed to cultural differences between countries (Vu et al., 2017). This finding is not consistent with the existing view that Latvia and Lithuania are both countries of an individualistic culture since Lithuanian teachers’ answers are more collectivism-oriented, whereas the Latvian teachers’ answers emphasize a more individualistic approach to reflecting on their professional work experiences.

## 5. Conclusion and implications

The results of the current study show that building a positive school climate and using empathy are of great importance in teachers’ professional work. Latvian and Lithuanian teachers claim that they invest in a positive school climate by building such aspects of their professional capability as coping with changes, acting autonomously, implementing leadership, and developing professionally. Specifically, Latvian teachers recognized relationships as an important aspect of promoting a positive school climate, whereas Lithuanian teachers spoke about creativity in their professional work and addressed whole-school aspects like values.

Latvian and Lithuanian teachers similarly recognize that they use empathy in their work through communication with a wide spectrum of partners (students, colleagues, administration, and parents) and in providing support to students. Regarding country-specific differences, Latvian teachers spoke about using empathy through appropriate classroom instruction, taking students’ perspectives, and changing their mindsets to adapt to their students’ needs. Lithuanian teachers provided more general statements about how they use empathy at work, emphasizing fundamental values (respect, dignity, and kindness) and the importance of collaboration with colleagues.

The results show a tendency toward a collectivistic culture in the Lithuanian teachers’ answers and a more individualistic attitude among Latvian teachers regarding contributing to a positive school climate and using empathy at work. What these differences mean for teachers’ contribution to a positive school climate and the use of empathy at work cannot be precisely answered using this dyad. However, if future research on this issue were carried out, it would contribute to our understanding of school psychology and education management in both countries.

Due to the qualitative design and the sample size, this study provides the opportunity to hear teachers’ voice to understand their needs in pre-service teacher education to provide knowledge and equip them with skills to build a positive learning environment and promote their students’ well-being. This study clearly shows that teachers recognize the need for training in how to establish and maintain positive relationships, sustain organizational values, apply positive discipline, and implement social–emotional learning through everyday teaching routines and formative assessment. This adjusts the implication that psychological readiness and methodological competence must be equally addressed in pre-service teacher education.

The content of curriculums for pre-service teacher training and education needs to be targeted to develop teachers’ awareness of their active role in every process at school. Teachers are not external experts teaching, observing, informing, disciplining, and assessing their students and must not just implement competency-based education but must also develop their competencies to become a tool, embodying their own values, rules, and teaching methods.

The findings of this research highlight that the following aspects need to be addressed in pre-service teacher education: (1) the central role of building relationships; (2) the development of teachers’ introspection and self-reflection to be capable of contributing personally to building a positive school climate and using empathy; and (3) having an awareness of and taking individual responsibility for sustaining institutional values.

### 5.1. Strengths and limitations

A strength of this research is that the sample represents different national regions and school types in two European countries. The qualitative study design provided the opportunity to hear teachers’ voices to gain an in-depth understanding of their experiences and perceptions of their contributions to creating a positive school climate and using empathy at work. The findings of this qualitative research also made several specific recommendations for developing pre-service teacher education curriculums in the future.

Due to the nature of this qualitative research, the results cannot be generalized as completely representing the societies and countries mentioned. One of limitations can be attributed to the fact that the teachers’ educational level was not addressed; however, the minimum pre-service education of teachers in Latvia and Lithuania is different. The gender distribution in the current sample was disproportional; nevertheless, it represents a gender distribution typical of the teacher populations in Latvia and Lithuania. Due to this, there was no possibility of addressing gender differences in the teachers’ answers. It is also possible that in a focus group environment, some teachers could not express their opinions in the same way as in an individual interview out of fear or intimidation of the majority opinion.

## Data availability statement

The datasets presented in this article can be made available upon request by contacting BM and VŽ. Requests to access the datasets should be directed to baiba.martinsone@lu.lv and vilma.zydziunaite@vdu.lt.

## Ethics statement

The studies involving human participants were reviewed and approved by Committee for Educational Research Ethics, Educational Research Institute, Vytautas Magnus University (February 17, 2022, Protocol No. 5) and from the Research Board of Vytautas Magnus University (March 1, 2022, Protocol No. 17). The participants provided their written informed consent to participate in this study.

## Author contributions

BM: key contribution to arranging the research in Latvia, collecting and analyzing data, and writing. VŽ: key contribution to designing the research, arranging the research in Lithuania, collecting and analyzing data, and writing. All authors contributed to the article and approved the submitted version.

## Funding

This study was conducted within the EU-funded Erasmus+ KA3 research project “Teaching to Be: Supporting Teachers Professional Growth and Wellbeing in the Field of Social and Emotional Learning” (No. 626155-EPP-1-2022-2-LT-EPPKA3-PI-POLICY).

## Conflict of interest

The authors declare that the research was conducted in the absence of any commercial or financial relationships that could be construed as a potential conflict of interest.

## Publisher’s note

All claims expressed in this article are solely those of the authors and do not necessarily represent those of their affiliated organizations, or those of the publisher, the editors and the reviewers. Any product that may be evaluated in this article, or claim that may be made by its manufacturer, is not guaranteed or endorsed by the publisher.
